# Feeding on rapid cold hardening *Ambrosia artemisiifolia* enhances cold tolerance of *Ophraella communa*

**DOI:** 10.3389/fpls.2023.1114026

**Published:** 2023-07-17

**Authors:** Zhenqi Tian, Chao Ma, Yan Zhang, Hongsong Chen, Xuyuan Gao, Jianying Guo, Zhongshi Zhou

**Affiliations:** ^1^ State Key Laboratory for Biology of Plant Diseases and Insect Pests, Institute of Plant Protection, Chinese Academy of Agricultural Sciences, Beijing, China; ^2^ National Nanfan Research Institute, Chinese Academy of Agricultural Sciences, Sanya, China; ^3^ Guangxi Key Laboratory for Biology of Crop Diseases and Insect Pests, Institute of Plant Protection, Guangxi Academy of Agricultural Sciences, Nanning, China

**Keywords:** cold tolerance, cryoprotectant, *Ambrosia artemisiifolia*, *Ophraella communa*, collaborative adaptation, trophic effects

## Abstract

Low temperatures greatly influence newly introduced species, and increased cold tolerance can facilitate their establishment in new environments. The invasive alien species *Ambrosia artemisiifolia* is distributed at high latitudes and altitudes, where it suffers more from cold stress than it would at low latitudes or altitudes. Whether cold stress influences the accumulation of cryoprotectants and cold tolerance in *A. artemisiifolia*, and further influences the cold tolerance of its biological control agent, *Ophraella communa*, through feeding remain unknown. We investigated the levels of cryoprotectants and metabolic changes in *A. artemisiifolia.* We found that the level of total sugar, trehalose, proline, and other cold responsible metabolites increased in *A. artemisiifolia* after rapid cold-hardening (RCH) treatment, when compared to normal plants. These indicated that RCH treatment could improve the cold-hardiness of *A. artemisiifolia*. We then investigated the levels of cryoprotectants and metabolic changes in *O. communa*. We found that *O. communa* fed on RCH-treated *A. artemisiifolia* had higher levels of total sugar, trehalose, proline, glycerol, lipid, lower water content, lower super-cooling point, and increased cold tolerance compared to *O. communa* fed on normal *A. artemisiifolia*. This suggested that *O. communa* fed on cold-hardened *A. artemisiifolia* could increase its cold tolerance. Results showed a trophic transmission in insect cold tolerance. Our study enriches the theoretical basis for the co-evolution of cold tolerance in invasive and herbivorous insects.

## Introduction

The establishment of introduced species in northern regions is limited by the low temperatures at high latitudes or altitudes. However, some alien plants and insects can overcome cold stress and adapt to low temperatures (Carbonell et al., 2021; Tian et al., 2022), which results in an expansion of their distribution. This cold tolerance of plants and insects has been extensively studied, and some inter-community mechanisms have been described, indicating that collaborative adaptation may exist in plant–herbivorous insect interactions.

In autumn, plants suffer from cold stress, and low temperatures can cause cell damage and osmotic impairment. Plants have evolved complex mechanisms to respond to cold stress (Ding et al., 2019), including morphological, molecular, biochemical, and physiological adaptations. Physiological reactions play an important role in plant cold tolerance. Plants can, for instance, accumulate large soluble saccharides, polyhydric alcohols, amino acids, and other low molecular weight compounds in autumn, such as trehalose and proline (Ding et al., 2019). These compounds could be termed as cryoprotectants, they function in membrane and protein stabilization (Tarkowski and Vanden Ende, 2015). The higher content of cold-tolerance compounds in plants in autumn can result in stronger cold tolerance than in summer (Rütten and Santarius, 1992).

Similar to plants, insects also have strategies for resisting cold damage. Many factors influence the cold tolerance of insects, such as geographic variation (Wang et al., 2010), seasonal change (Feng et al., 2016; Noh et al., 2017; Hou et al., 2021), host variety (Morey et al., 2016; Mutamiswa et al., 2020), developmental stage (Zhang et al., 2021), and diapause (Ciancio et al., 2021). These factors could regulate the physiological changes of insects, physiological mechanism plays an important role in insect cold tolerance. Insects usually reduce their body water content and accumulate high levels of cryoprotectants, such as sugars, polyhydric alcohols, amino acids, and other small molecular weight compounds. These accumulations could stabilize membranes and proteins (Yancey, 2005; Denlinger and Lee, 2010; Teets and Denlinger, 2013; Toxopeus and Sinclair, 2018). Ultimately, insects exhibit a strong super-cooling capacity to against cold injury.

Host plant or diet can positively affect cold tolerance, it mainly depends on nutrition in insect host or dietary composition (Shreve et al., 2007; Koštal et al., 2011; Colinet and Renault, 2014; Li et al., 2014; Koštal et al., 2016; Littler et al., 2021). It has been found that an increased concentration of proline (Koštal et al., 2011), arginine (Koštal et al., 2011; Koštal et al., 2016), cholesterol (Shreve et al., 2007), and live yeast (Colinet and Renault, 2014) in the larval diet of fruit flies could increase concentrations of dietary supplements in bodies of fruit flies, then further dramatically increased cold tolerance of the fruit flies. The increase in cold tolerance caused by dietary supplements can be transmitted across trophic levels in the parasitoid *Nasonia vitripennis* (Li et al., 2014). Whether similar patterns exist or not between plants and insect herbivores is still unknown, though the cold tolerance of insect herbivores are associated with their host plants (Liu et al., 2007; Liu et al., 2009; Kleynhans et al., 2014; Morey et al., 2016).

*Ambrosia artemisiifolia* is a noxious invasive alien weed that can cause allergic symptoms in humans and is harmful to the environment (Smith et al., 2013). It was first introduced in China in the 1930s, where it is now distributed in 23 provinces, from Guangxi to Heilongjiang province (Zhou et al., 2015). *Ophraella communa* is an effective biological control agent for *A. artemisiifolia* (Guo et al., 2011). It originated in North America and was occasionally found in Nanjing, China, in 2001 (Meng and Li, 2005). It has achieved great success in managing *A. artemisiifolia* in China and Europe (Zhou et al., 2015; Schaffner et al., 2020). Although this species is mainly distributed in southern China, in 2012 it was introduced to Beijing (39.98°N, 115.97°E), where it has managed to survive the cold winters and establish a stable population (Tian et al., 2020). This suggests that the species adapted to the local environment and improved its cold tolerance. Tian et al. (2022) showed that the individuals of Beijing population accumulate high levels of cryoprotectants and energy reserves. Based on the effect of trophic transmission on insect cold tolerance (Li et al., 2014), we hypothesized that the high accumulations of cryoprotectants or energy reserves in *O. communa* were associated with the high accumulations of cryoprotectants of the local *A. artemisiifolia* in Beijing, and the cold tolerance of *O. communa* could be improved by feeding on cold-hardened *A. artemisiifolia*.

In this study, we tested the change in the content of cold-responsive physiological activators in *A. artemisiifolia* after cold stress. We then detected the super-cooling point (SCP) and changes of physiological metabolism of *O. communa* after feeding on cold-hardened *A. artemisiifolia*. To further study whether certain cold-responsible minute matter trace matter was transferred from *A. artemisiifolia* to *O. communa*, the comparative metabolomes of the above-treated *A. artemisiifolia* and *O. communa* were tested. We found that rapid cold hardening (RCH) increased the physiological levels of cold tolerance in *A. artemisiifolia* and further increased the cold tolerance of *O. communa* by feeding. We confirmed the trophic transmission of cold tolerance between plants and insect herbivores. This result has enriched the insect cold tolerance theory and is valuable for improving the local adaptation of introduced biological control agents of plant pests.

## Materials and methods

### Host plants and insects

*Ambrosia artemisiifolia* seeds were collected from fields at Langfang Experimental Station of the Chinese Academy of Agricultural Sciences (CAAS), Langfang, Hebei Province, China (39°N, 116°E) in October 2019 and stored at 4°C. Two *A. artemisiifolia* plants were grown in plastic pots (10 × 10 × 8 cm) without fertilizer.

*Ophraella communa* adults were collected from *A. artemisiifolia* plants in fields of Laibin City, Guangxi Zhuang Autonomous Region, China (23.62°N, 109.37°E), in late June 2020. They were then reared in cages (40 × 60 cm) in a laboratory at 26 ± 1°C and 70 ± 5% relative humidity (RH) with a photoperiod of 14L:10D at the Langfang Experimental Station of CAAS.

### Rapid cold-hardening treatment of *Ambrosia artemisiifolia*

Whole *A. artemisiifolia* plants were exposed for 48 h to five temperatures ranging from 20°C to 8°C in 3°C increments under a short photoperiod of 10L:14D. Temperature gradients were set to simulate the temperature before leaf senescence of *A. artemisiifolia* in the autumn at different latitudes. The control group (CK) was maintained at 25°C under a long photoperiod of 14L:10D. After RCH treatment, leaves were cut out, immediately frozen in liquid nitrogen, and stored at −80°C for further extraction of metabolites and measurement of total sugar, trehalose, and proline content. Treatment under 14°C was selected to sequence metabolome, because this temperature is close to average temperatures in October in Beijing. Simultaneously, part of the leaves were cut out for further *O. communa* adult rearing.

### Feeding treatment for *Ophraella communa* adults

Newly emerged *O. communa* adults were collected from cages and identified as males and females, respectively. Treatment groups were set as individuals fed on RCH *A. artemisiifolia* leaves, and a control group was set as individuals fed on *A. artemisiifolia* leaves from above CK. The petioles of detached leaves were covered with wet cotton to keep the leaves fresh. The leaves were replaced every 24 h. These *O. communa* adults were reared in a growth chamber at 26 ± 1°C and 70 ± 5% RH with a photoperiod of 14L:10D. After continuous feeding for 3 days, *O. communa* became sexually mature. Some of them were used to measure the super-cooling point, while others were frozen in liquid nitrogen and stored at −80°C until analysis of metabolites and measurement of water, total sugar, trehalose, glycerol, lipid, and proline content. Be same as plant, beetles that fed on 14°C treated *A. artemisiifolia* leaves were selected to sequence metabolome.

### Measurement of total sugar, trehalose, and proline content in *Ambrosia artemisiifolia*

Sugar, trehalose, and proline are the main physiological accumulations in plants that adapt to cold stress (Krasensky and Jonak, 2012; Tarkowski and Vanden Ende, 2015), and are widely used to study cold tolerance in plants. Therefore, these three physiological levels were chosen for measurement in this study. The leaves were stored at −80°C.

For total sugar content measurement, *A. artemisiifolia* leaves were ground in a mortar, and 0.1 g leaves were weighed and transferred into a 1.5-mL centrifuge tube. Distilled water (1.5 mL) was then added and heated for 10 min at 100°C. After cooling, the samples were centrifuged at 5,000 rpm for 10 min at 25°C. Then, 1.0 mL of upper solution was transferred to a 10-mL centrifuge tube. Four milliliters of 0.20% anthrone-sulfuric acid reagent was added to the solutions and mixed, and the tube was heated for 10 min in a boiling water bath, cooled with running water, and equilibrated for 20 min. The zero point was set using a blank tube with the solution, and absorption at 620 nm was measured and recorded.

Trehalose content measurement, *A. artemisiifolia* leaves were ground in a mortar, and 0.1 g leaves were weighed and transferred into a 1.5-mL centrifuge tube. Distilled water (1.5 mL) was then added and heated for 10 min at 100°C. After cooling, the samples were centrifuged at 5,000 rpm for 10 min at 25°C. Then, 1.0 mL of upper solution was transferred to a 10-mL centrifuge tube. After 0.5 mL 1.50% H_2_SO_4_ solution was added to the solutions, the tube was heated in a 90°C-water bath for 10 min. After cooling, 0.5 mL 30% KOH solution was added to the mixture and heated again for 10 min. Four milliliters of 0.20% anthrone-sulfuric acid reagent was added to the solutions and mixed, and the tube was heated for 10 min in a boiling water bath, cooled with running water, and equilibrated for 20 min. The zero point was set using a blank tube with the solution, and absorption at 620 nm was measured and recorded.

For proline content measurement, *A. artemisiifolia* leaves were ground in a mortar, and 0.1 g leaves were weighed and transferred into a 1.5-mL centrifuge tube. Then, 500 mL of sulfosalicylic acid was added to the tube, heated for 10 min at 100°C, and centrifuged at 10,000 ×g for 10 min at 25°C. After cooling, 0.25 mL supernatant, glacial acetic acid, and ninhydrin were individually added to a 2-mL centrifuge tube. The mixture was incubated for 30 min at 100°C, with shaking every 10 min. After cooling, 0.5 mL toluene was added, and the mixture was shaken for 30 min for proline extraction. We selected 0.2 mL of the upper solution to detect absorption using a quartz micro cuvette at 520 nm.

All measurements were conducted for three replicates.

### Measurement of super-cooling point of *Ophraella communa*

The SCP is widely used to measure insect cold tolerance (Zhou et al., 2011; Li et al., 2021; Tian et al., 2022). SCP was defined as the lowest temperature recorded before a sudden increase in temperature, caused by the release of the latent heat of crystallization. Thermocouples were placed in contact with the cuticle of individual beetles, and then in a −25°C freezer at a cooling rate of approximately 1°C per minute (Zhou et al., 2011; Tian et al., 2022). Three replicates with 10–15 individuals each were considered.

### Measurement of water, total sugar, trehalose, glycerol, lipid, and proline contents

For water content measurement, the wet weight (WW) of individual beetles was determined. The beetles were then placed in a 1.5-mL centrifuge tube and dried for 48 h at 60°C. After drying, the samples were weighed again and dry weight (DW) was recorded. The percentage of water was calculated as [(WW − DW)/WW] × 100. Total sugar, trehalose, glycerol, lipid, and proline contents were measured as described by Tian et al. (2022). All of the above measurements were conducted using three technique replicates of three beetles each.

### Effect of feeding on RCH-treated *Ambrosia artemisiifolia* on the development of *Ophraella communa*

To determine whether feeding on RCH-treated *A. artemisiifolia* was harmful to *O. communa*, the development of *O. communa* was tested. The first instar larvae of *O. communa* fed on RCH-treated *A. artemisiifolia* were selected as a treatment group, and those fed on common temperature (26°C)-treated *A. artemisiifolia* was the control treatment (CK). They were reared in a growth chamber at 25°C under a long photoperiod of 14L:10 D. Fresh *A. artemisiifolia* leaves from the RCH and CK treatments were replaced every 24 h. Molting, pupation, and emergence were recorded every 24 h. Five replicates were used, and ten group-reared individuals were replicated.

### Statistical analysis

Differences in total sugar, trehalose, and proline content in *A. artemisiifolia* among different RCH treatments, and in water content, total sugar, trehalose, proline, glycerol, lipid content, and SCP of *O. communa* among different treatments were analyzed using Tukey’s honest significant difference (HSD) tests with SAS 8.1 (SAS Institute, Cary, NC, USA). The difference in development time of different stages of *O. communa* between treatment (fed on 14°C-treated *A. artemisiifolia*) and CK (fed on normal *A. artemisiifolia*) was analyzed by *t*-test with SAS 8.1.

## Results

### Effect of RCH on total sugar, trehalose, and proline content in *Ambrosia artemisiifolia*

**Figure 1:**
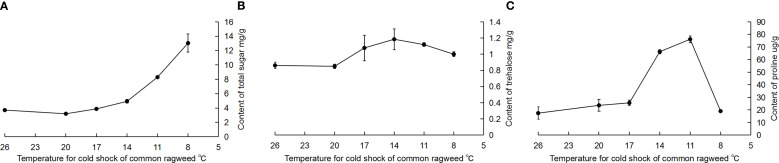
Effect of cold-hardening on the physiological contents of *Ambrosia artemisiifolia*. **(A)** total sugar; **(B)** trehalose; **(C)** proline. Different lowercase letters represent significant difference among treatments (*P* < 0.05, Tukey HSD test). The data are showed as mean ± SE.

The total sugar content was significantly increased after RCH in *A. artemisiifolia* when compared with the common group (*F* = 67.40, *df* = 5, 15, *P* < 0.05) (**Figure 1A**). Trehalose content increased slightly, but the difference was not significant (*F* = 0.77, *df* = 5, 18, *P* = 0.58) (**Figure 1B**). Proline content was significantly increased and was highest when RCH was at 11°C; however, it sharply decreased when RCH was at 8°C (*F* = 71.56, *df* = 5, 16, *P* < 0.05) (**Figure 1C**).

### Change of cold tolerance in *Ophraella communa* when feeding different treatment on *Ambrosia artemisiifolia*

**Figure 2:**
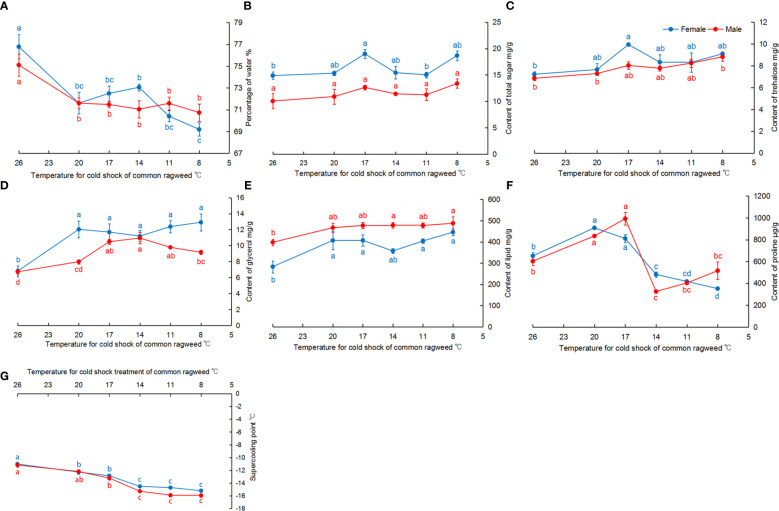
Physiological contents and the super-cooling point (SCP) of adult *Ophraella communa* fed on cold-treated *Ambrosia artemisiifolia*. **(A)** water content; **(B)** total sugar content; **(C)**: trehalose content; **(D)** glycerol content; **(E)**: lipid content; **(F)** proline content; **(G)** SCP.

Cold tolerance of *O. communa* significantly changed after they were fed RCH-treated *A. artemisiifolia*. Compared with CK, the percentage of water in *O. communa* was significantly reduced (female: *F* = 11.94, *df* = 5, 18, *P* < 0.05; male: *F* = 5.21, *df* = 5, 18, *P* < 0.05) (**Figure 2A**). The trends of total sugar and trehalose were similar, and their content increased in the treatment groups. Their contents in female adults significantly changed among treatments (total sugar: *F* = 5.66, *df* = 5, 12, *P* < 0.05; trehalose: *F* = 3.60, *df* = 5, 12, *P* < 0.05) and were highest at 17°C (**Figures 2B, C**). In male adults fed RCH-treated *A. artemisiifolia*, the total sugar content increased, but not significantly (*F* = 1.36, *df* = 5, 12, *P* = 0.30), whereas the trehalose content significantly increased (*F* = 5.50, *df* = 5, 12, *P* < 0.05) (**Figures 2B, C**). The glycerol content in adults from the treatment groups significantly increased (female: *F* = 6.17, *df* = 5, 18, *P* < 0.05; male: *F* = 16.37, *df* = 5, 18, *P* < 0.05) (**Figure 2D**). Significant differences were also found in lipid content among the different treatments (female: *F* = 5.04, *df* = 5, 18, *P* < 0.05; male: *F* = 3.18, *df* = 5, 18, *P* < 0.05) (**Figure 2E**). The proline content in adults increased and decreased in *O. communa* fed on *A. artemisiifolia* treated with RCH from 26°C to 17°C and 14°C to 8°C, respectively (female: *F* = 86.92, *df* = 5, 12, *P* < 0.05; male: *F* = 33.58, *df* = 5, 12, *P* < 0.05) (**Figure 2F**). The SCP of adults in the treatment groups was significantly reduced when compared to CK (female: *F* = 35.35, *df* = 5, 208, *P* < 0.05; male: *F* = 39.84, *df* = 5, 193, *P* < 0.05) (**Figure 2G**). When adults were fed RCH-treated *A. artemisiifolia*, their SCP was reduced to −15 °C (**Figure 2G**).

### Effect of RCH on the metabolome of *Ambrosia artemisiifolia*

**Figure 3:**
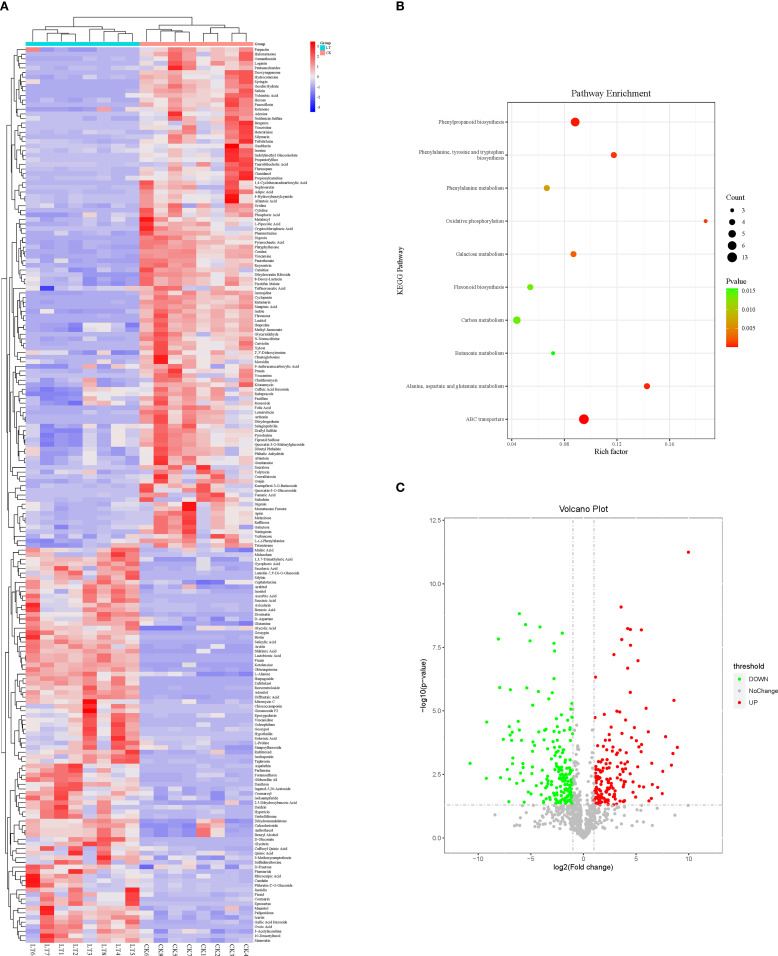
**(A)** Heat map of metabolites in *Ambrosia artemisiifolia* from rapid cold-hardening- (RCH; 14 °C) and common temperature-treated (26 °C) samples; **(B)** scatter plot for KEGG pathway enrichment of differentially expressed metabolites in RCH- and temperature-treated *A. artemisiifolia*; **(C)** differentially expressed metabolites in RCH- and temperature-treated *A. artemisiifolia*.

RCH significantly affected the metabolism of *A. artemisiifolia*. Significant metabolic changes in *A. artemisiifolia* between RCH and CK were observed for 195 chemical compounds (**Figure 3**), including flavonoids (silybin, luteolin-7,3’-di-O-glucoside, and diosmetin), organic acids (salicylic acid, succinic acid, benzoic acid, amino acids, and derivatives, such as L-proline, glutamine, and alanine), and carbohydrates and alcohols (mannitol, D-fructose, and ribitol), which are linked to different metabolic pathways. The increased metabolic changes were mainly enriched in ABC transporters, phenylpropanoid biosynthesis, alanine, aspartate, and glutamate metabolism, and galactose metabolism.

### Effect of feeding on the metabolome of *Ophraella communa*

**Figure 4:**
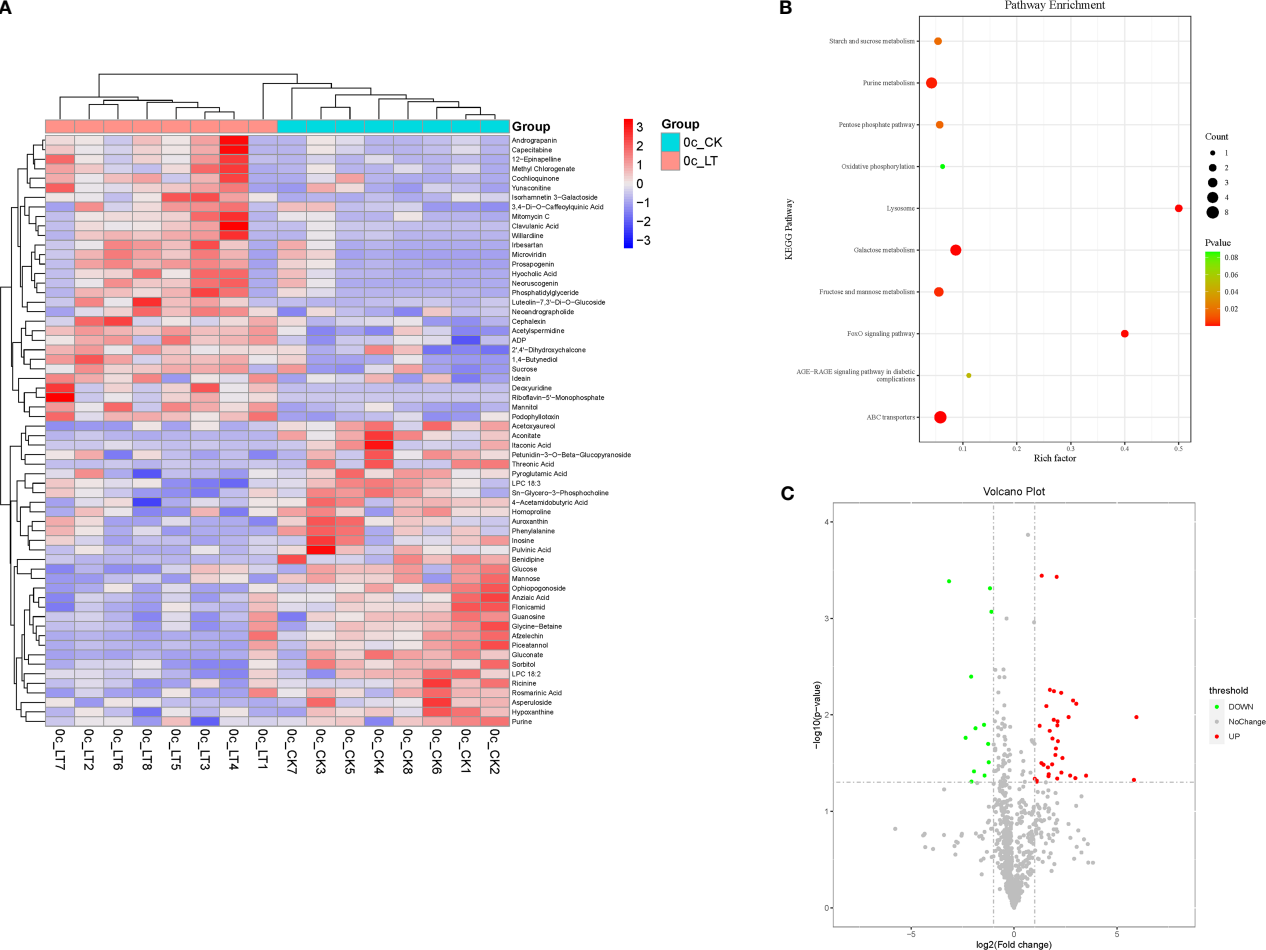
**(A)** Heat map of metabolites identified in adult *Ophraella communa* from rapid cold-hardening- (RCH; 14 °C) and common temperature-treated (26 °C) samples; **(B)** scatter plot for KEGG pathway enrichment of differentially expressed metabolites in adult *O. communa* fed with RCH- and temperature-treated *Ambrosia artemisiifolia*; **(C)** differentially expressed metabolites in adult *O. communa* fed with RCH- and temperature-treated *A. artemisiifolia*.

Feeding on RCH-treated *A. artemisiifolia* significantly affected the metabolism of *O. communa*. Significant metabolic differences were found between RCH and CK *O. communa* for 62 chemical compounds (**Figure 4**), including flavonoids (luteolin-7,3’-di-O-glucoside, and isorhamnetin 3-galactoside), carbohydrates and alcohols (mannitol and sucrose), and nucleic acids and derivatives (deoxyuridine, flavin nucleotides, and ADP), which are linked to different metabolic pathways. The increased metabolic changes were mainly associated with ABC transporters, galactose metabolism, and purine metabolism.

### Effect of feeding on the development of larvae of *Ophraella communa*

**Figure 5:**
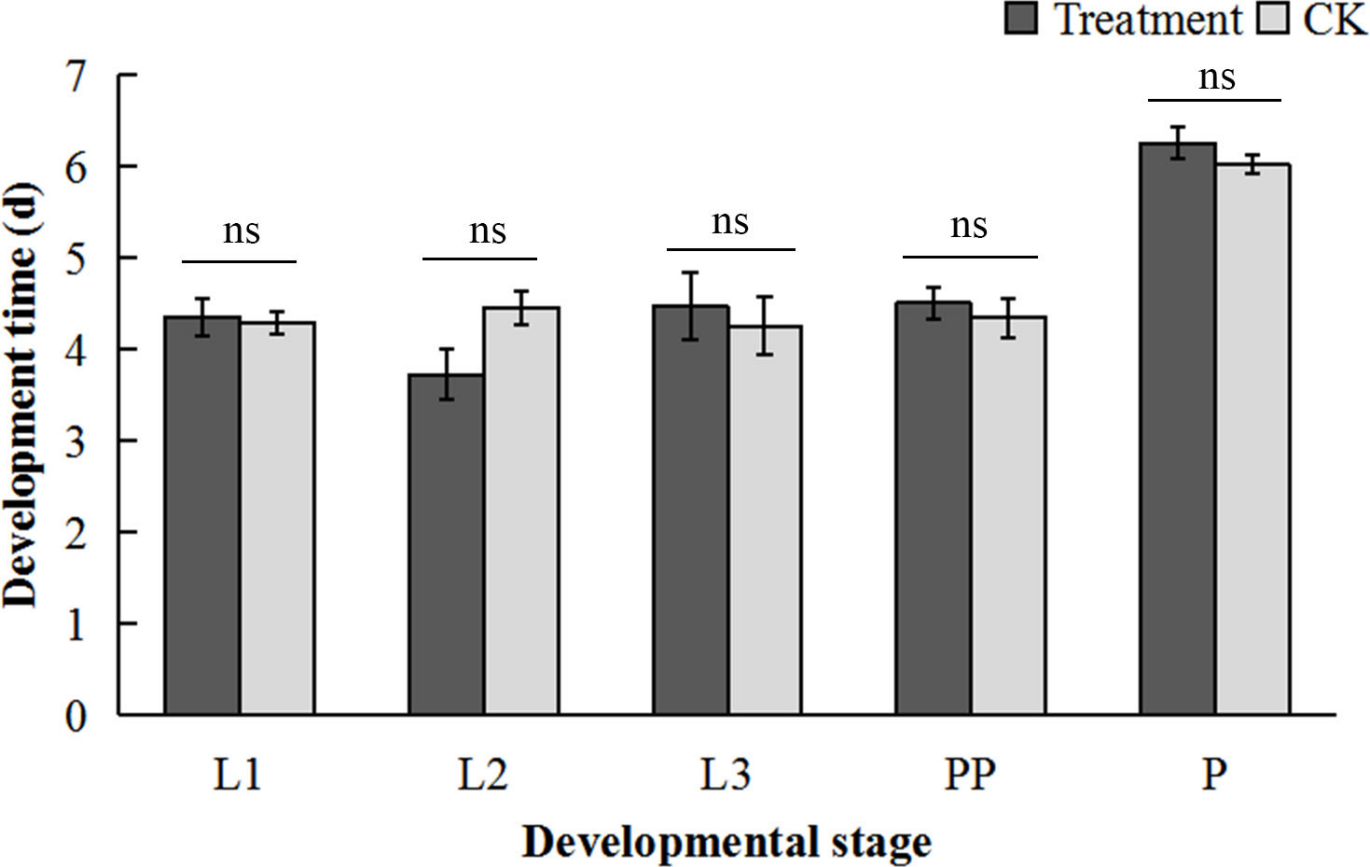
Effect of cold-hardened *Ambrosia artemisiifolia* on the development of *Ophraella communa*. L1, first-instar larvae; L2, second-instar larvae; L3, third-instar larvae; PP, prepupae; P, pupae. Treatment, fed on cold-hardened *A. artemisiifolia*; CK, fed on normal *A. artemisiifolia*. (*t*-test, *P* < 0.05).

No significant differences were observed in the developmental duration of each stage of *O. communa* between CK (fed on normal *A. artemisiifolia*) and RCH treatment (fed on 14°C-treated *A. artemisiifolia*) (L1: *t* = 0.26, *P* = 0.80; L2: *t* = 2.19, *P* = 0.06; L3: *t* = 0.44, *P* = 0.67; PP: *t* = 0.58, *P* = 0.58; P: *t* = 1.18, *P* = 0.27) (**Figure 5**).

## Discussion

Invasion by alien species is a form of adapting to new environments, and these species have the ability to establish themselves in a new environment. The low temperatures at high latitudes or altitudes are a barrier for these species. However, the cold stress also limits the introduction of natural enemies to manage invasive alien species. Feeding-mediated trophic transmission likely promotes cold adaptation in insects. Currently, whether the cold-hardiness of host plants can regulate the cold tolerance of insect herbivores by feeding is still unknown.

In this study, the contents of total sugar, trehalose, and proline in *A. artemisiifolia* increased after RCH treatment, and the levels of important small molecular weight compounds such as alanine, ribosol, mannitol, and fructose were also increased. These results suggest that the *A. artemisiifolia* distributed from southern to northeastern China suffer from different levels of low-temperature stress, have various physiological accumulations, and show a variance in cold tolerance. However, the dynamics of different cold-responsive physiological substances in *A. artemisiifolia* varied when cold-hardening at different temperatures was tested. The total sugar content significantly increased, whereas trehalose content did not. This suggests that the content of some other sugars in *A. artemisiifolia*, such as fructose, was significantly increased by metabolome sequencing (**Figure 3**). The cold-hardiness of *A. artemisiifolia* was improved, reflecting the accumulation and upregulation of cytoprotectants (antioxidant defense). Flavonoids in plants function as antioxidants, which play an important role in cold tolerance (Korn et al., 2008; Schulz et al., 2016). For instance, silymarin scavenges ROS and maintains cell mobility. These results suggest that RCH can enhance the cold-hardiness of *A. artemisiifolia*.

Although total sugar, trehalose, and proline contents play key roles in enhancing insect cold tolerance, other low-molecular-weight compounds such as sorbitol or mannitol also respond to cold stress. Therefore, sequencing the metabolome to analyze the metabolic changes before and after treatment remains necessary. In this study, the level of main cryoprotectants increased in RCH-treated *A. artemisiifolia* and in *O. communa* fed on cold-hardened hosts. Compared with those feeding on common *A. artemisiifolia*, *O. communa* fed on RCH-treated *A. artemisiifolia* had higher levels of mannitol and phosphatidyl triglycerides. Mannitol is one of the most important cryoprotectants, and its content and insect cold tolerance are positively correlated (Sømme, 1969; Teets and Denlinger, 2013). Phosphatidyl triglycerides are a basic component of the plasmolemma and effectively maintain plasma membrane fluid and stability. The flavin protein cogroup, flavin nucleotide, and adenosine diphosphate are associated with respiratory chain electron transport, which indicates that metabolic rates changed and provided more energy in *O. communa* feeding on RCH-treated *A. artemisiifolia*. Most importantly, the levels of mannitol and luteolin-7,3’-di-O-glucoside increased in both RCH-treated *A. artemisiifolia* and *O. communa*. This probably suggests a trophic transmission in cold tolerance of insects.

Proline is an important cryoprotectant for both plants and insects. However, in the present study, the changes were different in *A. artemisiifolia* and *O. communa*. Regarding the content of proline in *A. artemisiifolia*, similar patterns occur in *Carex* spp., *Duranta erecta* ‘Golden Edge’, and *Brugmansia arborea* (Ye et al., 2017; Liu et al., 2019). This suggests that the dynamics of proline content can be divided into two phases: accumulation and degradation. When temperatures reduce, proline is actively accumulated immediately to regulate osmotic potential, stabilize cells and macromolecules, and further resist the more intense low-temperature stress. Surprisingly, the proline content in *O. communa* did not gradually increase when *O. communa* was fed *A. artemisiifolia* at lower temperatures (**Figure 2**). Although the proline content in *A. artemisiifolia* was higher at 14°C and 11°C, it did not increase after *O. communa* was fed. A similar trend was observed for *Sarcophaga crassipalpis*; after increasing the proline in the diet of *S. crassipalpis*, the content of proline did not increase in diapausal flies, but increased proline content in the fly (Li et al., 2014). Li et al. (2014) suggested that cryoprotectants more important than proline accumulate in diapausal *S. crassipalpis*. Therefore, we speculate that *O. communa* accumulated more important cryoprotectants, such as mannitol or other substances, resulting in a low proline level.

In the present study, we confirmed trophic transmission of insect cold tolerance. Insect cold tolerance resulting from trophic transmission has been previously reported (Koštal et al., 2011; Li et al., 2014), but from studies using supplements in artificial diets, usually with was a single component or at a concentration higher than the natural intake. In the present study, the physiological changes (including up- and down-regulation) were observed under semi-natural conditions and considering the effect of RCH on *A. artemisiifolia*, thus providing results much more closer to nature. Moreover, not all amino compounds are positive diet additives for improving insect cold tolerance, as some may be poisonous (Koštal et al., 2016), but the RCH-treated *A. artemisiifolia* herein used was harmless to *O. communa* (**Figure 5**).

Host plants can influence the cold tolerance of insects (Liu et al., 2007; Liu et al., 2009; Kleynhans et al., 2014; Morey et al., 2016). Feeding links insect cold tolerance to host plant quality or diet components. *Helicoverpa armigera* fed on different host plants, such as corn, cotton, or tobacco, showed variance in cryoprotectant accumulation and further cold tolerance (Liu et al., 2007; Liu et al., 2009). Nutrient levels vary in different host plants, which explains the differences in insect cold tolerance. In our study, the nutrient levels in *A. artemisiifolia* varied under different temperatures, which contributed to the improvement in insect cold tolerance. This shows that this approach is effective to increase the cold tolerance of insect herbivores and improve the local adaptation of introduced biological control agents of plant pests.

In conclusion, low temperatures appear to influence the physiological levels of plants, and the feeding of insect herbivores further influences their physiological levels and increases their cold tolerance. This indicates the trophic transmission of cold tolerance between plants and insect herbivores. Therefore, insects are suggested to accumulate cryoprotectants in through their own metabolism or from their feeding to resist cold stress.

## Data availability statement

The raw data supporting the conclusions of this article will be made available by the authors, without undue reservation.

## Author contributions

ZZ conceived and designed research. ZT, CM and YZ conducted experiments. CM, YZ, HC, XG, and JG analyzed data. ZZ and ZT wrote the manuscript. All authors contributed to the article and approved the submitted version.

## References

[B1] Carbonell J. A. Wang Y. J. Stoks R. (2021). Evolution of cold tolerance and thermal plasticity in life history, behaviour and physiology during a poleward range expansion. J. Anim. Ecol. 90, 1666–1677. doi: 10.1111/1365-2656.13482 33724470

[B2] Ciancio J. J. Turnbull K. F. Gariepy T. D. Sinclair B. J. (2021). Cold tolerance, water balance, energetics, gas exchange, and diapause in overwintering brown marmorated stink bugs. J. Insect Physiol. 128, 104171. doi: 10.1016/j.jinsphys.2020.104171 33227277

[B3] Colinet H. Renault D. (2014). Dietary live yeast alters metabolic profiles, protein biosynthesis and thermal stress of *Drosophila melanogaster* . Comp. Biochem. Physiol. A. Mol. Integr. Physiol. 170, 6–14. doi: 10.1016/j.cbpa.2014.01.004 24434805

[B4] Denlinger D. L. Lee R. E. Jr (Eds.) (2010). Low temperature biology of insects (New York: Cambridge University Press), pp 15–19.

[B5] Ding Y. Shi Y. Yang S. (2019). Advances and challenges in uncovering cold tolerance regulatory mechanisms in plants. New Phytol. 222, 1690–1704. doi: 10.1111/nph.15696 30664232

[B6] Feng Y. Xu L. Li W. Xu Z. Cao M. Wang J.. (2016). Seasonal changes in supercooling capacity and major cryoprotectants of overwintering Asian longhorned beetle (*Anoplophora glabripennis*) larvae. Agr. For. Entomol. 18, 302–312. doi: 10.1111/afe.12162

[B7] Guo J. Y. Zhou Z. S. Zheng X. W. Chen H. S. Wan F. H. Luo Y. H. (2011). Control efficiency of leaf beetle, *Ophraella communa*, on the invasive common ragweed, *Ambrosia artemisiifolia*, at different growing stages. Biocontrol Sci. Techn. 21, 1049–1063. doi: 10.1080/09583157.2011.603823

[B8] Hou Z. Dong Y. Shi F. Xu Y. Ge S. Tao J.. (2021). Seasonal shifts in cold tolerance and the composition of the gut microbiome of dendroctonus valens LeConte occur concurrently. Forests 12, 888. doi: 10.3390/f12070888

[B9] Kleynhans E. Conlong D. E. Terblanche J. S. (2014). Host plant-related variation in thermal tolerance of *Eldana saccharina* . Entomol. Exp. Appl. 150, 113–122. doi: 10.1111/eea.12144

[B10] Korn M. Peterek S. Hans-Peter M. Heyer A. G. Hincha D. K. (2008). Heterosis in the freezing tolerance, and sugar and flavonoid contents of crosses between *Arabidopsis thaliana* accessions of widely varying freezing tolerance. Plant Cell Environ. 31, 813–827. doi: 10.1111/j.1365-3040.2008.01800.x 18284584 PMC2440548

[B11] Koštal V. Korbelovaí J. Poupardin R. Moos M. Šimek P. (2016). Arginine and proline applied as food additives stimulate high freeze tolerance in larvae of *Drosophila melanogaster* . J. Exp. Biol. 219, 2358–2367. doi: 10.1242/jeb.142158 27489218

[B12] Koštal V. Zahradničkova H. Šimek P. (2011). Hyperprolinemic larvae of thedrosophilid fly, *Chymomyza costata*, survive cryopreservation in liquid nitrogen. Proc. Natl. Acad. Sci. U. S. A. 108, 13041–13046. doi: 10.1073/pnas.1107060108 21788482 PMC3156168

[B13] Krasensky J. Jonak C. (2012). Drought, salt, and temperature stress-induced metabolic rearrangements and regulatory networks. J. Exp. Bot. 63, 1593–1608. doi: 10.1093/jxb/err460 22291134 PMC4359903

[B14] Li X. Li D. Zhang Z. Huang J. Zhang J. Hafeez M.. (2021). Supercooling capacity and cold tolerance of the south American tomato pinworm, *Tuta absoluta*, a newly invaded pest in China. J. Pest Sci. 94, 845–858. doi: 10.1007/s10340-020-01301-y

[B15] Li Y. Zhang L. Zhang Q. Chen H. Denlinger D. L. (2014). Host diapause status and host diets augmented with cryoprotectants enhance cold hardiness in the parasitoid *Nasonia vitripennis* . J. Insect Physiol. 70, 8–14. doi: 10.1016/j.jinsphys.2014.08.005 25158026

[B16] Littler A. S. Garcia M. J. Teets N. M. (2021). Laboratory diet influences cold tolerance in a genotype-dependent manner in *Drosophila melanogaster* . Comp. Biochem. Physiolo. A. Mol. Integr. Physiol. 257, 110948. doi: 10.1016/j.cbpa.2021.110948 33819503

[B17] Liu Z. Gong P. Heckel D. G. Wei W. Sun J. Li D. (2009). Effects of larval host plants on over-wintering physiological dynamics and survival of the cotton bollworm, *Helicoverpa armigera* (Hübner) (Lepidoptera: noctuidae). J. Insect Physiol. 55, 1–9. doi: 10.1016/j.jinsphys.2008.07.017 18761347

[B18] Liu Z. Gong P. Wu K. Wei W. Sun J. Li D. (2007). Effects of larval host plants on over-wintering preparedness and survival of the cotton bollworm, *Helicoverpa armigera* (Hübner) (Lepidoptera: noctuidae). J. Insect Physiol. 53, 1016–1026. doi: 10.1016/j.jinsphys.2007.05.005 17597144

[B19] Liu G. Y. Wang Q. Mao Z. X. Li X. J. Wang W. Li Y. (2019). Effects of natural low temperature on cold-resistance physiological indexes of three subtropical ornamental plants. Mol. Plant Breed. 17, 5136–5143. doi: 10.13271/j.mpb.017.005136

[B20] Meng L. Li B. P. (2005). Advances on biology and host specificity of the newly introduced beetle, *Ophraella communa* LeSage (Coleoptera: chrysomelidae), attacking *Ambrosia artemisiifolia* (Compositae) in continent of China. Chin. J. Bio. Control. 21, 65–69. doi: 10.16409/j.cnki.2095-039x.2005.02.001

[B21] Morey A. C. Venette R. C. Nystrom Santacruz E. C. Mosca L. A. Hutchison W. D. (2016). Host-mediated shift in the cold tolerance of an invasive insect. Ecol. Evol. 6, 8267–8275. doi: 10.1002/ece3.2564 27878094 PMC5108276

[B22] Mutamiswa R. Machekano H. Nyamukondiwa C. Chidawanyika F. (2020). Host plant-related responses on the thermal fitness of *Chilo partellus* (Swinhoe) (Lepidoptera: crambidae). Arthropod-Plant. Inte. 14, 463–471. doi: 10.1007/s11829-020-09762-9

[B23] Noh S. Everman E. R. Berger C. M. Morgan T. J. (2017). Seasonal variation in basal and plastic cold tolerance: adaptation is influenced by both long- and short-term phenotypic plasticity. Ecol. Evol. 7, 5248–5257. doi: 10.1002/ece3.3112 28770063 PMC5528237

[B24] Rütten D. Santarius K. A. (1992). Relationship between frost tolerance and sugar concentration of various bryophytes in summer and winter. Oecologia 91, 260–265. doi: 10.1007/BF00317794 28313467

[B25] Schaffner U. Steinbach S. Sun Y. Skjøth C. A. de Weger L. A. Lommen S. T.. (2020). Biological weed control to relieve millions from *Ambrosia allergies* in Europe. Nat. Commun. 11, 1–7. doi: 10.1038/s41467-020-15586-1 32317698 PMC7174423

[B26] Schulz E. Tohge T. Zuther E. Fernie A. R. Hincha D. K. (2016). Flavonoids are determinants of freezing tolerance and cold acclimation in *Arabidopsis thaliana* . Sci. Rep. 6, 34027. doi: 10.1038/srep34027 27658445 PMC5034326

[B27] Shreve S. M. Yi S. X. Lee R. E. (2007). Increased dietary cholesterol enhances cold tolerance in *Drosophila melanogaster* . Cryo. Lett. 28, 33–37. 17369960

[B28] Smith M. Cecchi L. Skjøth C. A. Karrer G. Šikoparija B. (2013). Common ragweed: a threat to environmental health in Europe. Environ. Int. 61, 115–126. doi: 10.1016/j.envint.2013.08.005 24140540

[B29] Sømme L. (1969). Mannitol and glycerol in overwintering aphid eggs. Norsk. Entomologisk. Tidsskrift. 16, 107–111.

[B30] Tarkowski Ł.P. Vanden Ende W. (2015). Cold tolerance triggered by soluble sugars: a multifaceted countermeasure. Front. Plant Sci. 6. doi: 10.3389/fpls.2015.00203 PMC4396355 25926837

[B31] Teets N. M. Denlinger D. L. (2013). Physiological mechanisms of seasonal and rapid cold hardening in insects. Physiol. Entomol. 38, 105–116. doi: 10.1111/phen.12019

[B32] Tian Z. Q. Chen G. M. Zhang Y. Ma C. Tian Z. Y. Gao X. Y.. (2022). Rapid evolution of *Ophraella communa* cold tolerance in new low-temperature environments. J. Pest Sci. 95, 1233–1244. doi: 10.1007/s10340-021-01461-5

[B33] Tian Z. Q. Ma C. Cui S. W. Zhou Z. S. (2020). *Ophraella communa* can establish population in the suburbs of Beijing, China. J. Environ. Entomol. 42, 1039–1040. doi: 10.3969/j.issn.1674-0858.2020.04.31

[B34] Toxopeus J. Sinclair B. J. (2018). Mechanisms underlying insect freeze tolerance. Biol. Rev. Camb. Philos. Soc 93, 1891–1914. doi: 10.1111/brv.12425 29749114

[B35] Wang X. H. Qi X. L. Kang L. (2010). Geographic differences on accumulation of sugars and polyols in locust eggs in response to cold acclimation. J. Insect Physiol. 56, 966–970. doi: 10.1016/j.jinsphys.2010.04.008 20416314

[B36] Yancey P. H. (2005). Organic osmolytes as compatible, metabolic and counteracting cytoprotectants in high osmolarity and other stresses. J. Exp. Biol. 208, 2819–2830. doi: 10.1242/jeb.01730 16043587

[B37] Ye Y. R. Wang W. L. Zheng C. S. Fu D. J. Liu H. W. (2017). Evaluation of cold resistance of four wild *Carex* species. Chin. J. Appl. Ecol. 28, 89–95. doi: 10.13287/j.1001-9332.201701.035 29749192

[B38] Zhang D. Zhao S. Wu Q. Li Y. Wu K. (2021). Cold hardiness of the invasive fall armyworm, *Spodoptera frugiperda* in China. J. Integr. Agr. 20, 764–771. doi: 10.1016/S2095-3119(20)63288-9

[B39] Zhou Z. S. Guo J. Y. Li M. Ai H. M. Wan F. H. (2011). Seasonal changes in cold hardiness of *Ophraella communa* . Entomol. Exp. Appl. 140, 85–90. doi: 10.1111/j.1570-7458.2011.01128.x

[B40] Zhou Z. S. Guo J. Y. Wan F. H. (2015). Review on management of *Ambrosia artemisiifolia* using natural enemy insects. Chin. J. Bio. Control. 31, 657–665. doi: 10.16409/j.cnki.2095-039x.2015.05.006

